# Sweating Rate and Sweat Sodium Concentration in Athletes: A Review of Methodology and Intra/Interindividual Variability

**DOI:** 10.1007/s40279-017-0691-5

**Published:** 2017-03-22

**Authors:** Lindsay B. Baker

**Affiliations:** Gatorade Sports Science Institute, 617 W. Main St., Barrington, IL 60010 USA

## Abstract

Athletes lose water and electrolytes as a consequence of thermoregulatory sweating during exercise and it is well known that the rate and composition of sweat loss can vary considerably within and among individuals. Many scientists and practitioners conduct sweat tests to determine sweat water and electrolyte losses of athletes during practice and competition. The information gleaned from sweat testing is often used to guide personalized fluid and electrolyte replacement recommendations for athletes; however, unstandardized methodological practices and challenging field conditions can produce inconsistent/inaccurate results. The primary objective of this paper is to provide a review of the literature regarding the effect of laboratory and field sweat-testing methodological variations on sweating rate (SR) and sweat composition (primarily sodium concentration [Na^+^]). The simplest and most accurate method to assess whole-body SR is via changes in body mass during exercise; however, potential confounding factors to consider are non-sweat sources of mass change and trapped sweat in clothing. In addition, variability in sweat [Na^+^] can result from differences in the type of collection system used (whole body or localized), the timing/duration of sweat collection, skin cleaning procedure, sample storage/handling, and analytical technique. Another aim of this paper is to briefly review factors that may impact intra/interindividual variability in SR and sweat [Na^+^] during exercise, including exercise intensity, environmental conditions, heat acclimation, aerobic capacity, body size/composition, wearing of protective equipment, sex, maturation, aging, diet, and/or hydration status. In summary, sweat testing can be a useful tool to estimate athletes’ SR and sweat Na^+^ loss to help guide fluid/electrolyte replacement strategies, provided that data are collected, analyzed, and interpreted appropriately.

## Introduction

During exercise, water and electrolytes are lost as a consequence of thermoregulatory sweating. In some situations, especially when exercise is prolonged, high-intensity, and/or in a hot environment, sweat losses can be sufficient to cause excessive water/electrolyte imbalances and impair performance [1–5]. It is well-established that sweating rate (SR) and sweat electrolyte concentrations can vary considerably as a result of many within- and between-athlete factors (i.e. natural or expected sources of variability); therefore, personalized fluid replacement strategies are recommended [2, 6, 7]. In accordance with these guidelines, many scientists and practitioners have conducted sweat tests with athletes [8–17]; however, many different methodologies have been used, which could be another source of (undesirable) variability in SR and sweat electrolyte concentrations. For example, sweat testing can be conducted using whole-body techniques or localized to a specific anatomical site. Furthermore, methods to measure sweat electrolyte concentration can vary in the type of collection system used, the timing/duration of sweat collection, skin cleaning procedure, sample storage/handling, and analytical technique. The use of invalid or inconsistent methods related to any of these factors can lead to significant background noise, errors, and/or misinterpretation of results [18, 19]. However, there are few reviews available on sweat testing athletes, particularly with respect to personalized fluid replacement and best practices in the field.

The primary purpose of this paper is to review the methodological considerations for sweat-testing athletes. In addition, this review will briefly discuss within and between-subject factors that impact the variability in SR and sweat composition. Although many electrolytes and other constituents are lost in sweat, this review will primarily focus on sweat sodium concentration ([Na^+^]) since it is the electrolyte lost in the greatest quantities and has the most significant impact on body fluid balance (through the effects of Na^+^ on fluid retention and plasma volume maintenance/restoration) [1]. Finally, based on the information gleaned from this literature review, recommendations regarding best practices for sweat testing in the field, as well as considerations for interpretation and practical application of results, will be proposed.

## Eccrine Sweat Glands and Thermoregulatory Sweating

During exercise, a large amount of heat is produced by the contracting muscles as a byproduct of metabolism, leading to body heat gain. In addition, if ambient temperature is greater than skin temperature (*T*_sk_), heat is transferred from the air to the body. The resultant increase in body core temperature (*T*_c_) is sensed by central and skin thermoreceptors and this information is processed by the preoptic hypothalamic region of the brain to stimulate sweating and cutaneous vasodilation to dissipate heat [20–22]. Evaporation of sweat is the primary avenue of heat loss during exercise. With sweating, heat is transferred from the body to water (sweat) on the surface of the skin. When this water gains sufficient heat, it is converted to water vapor, thereby removing heat from the body (580 kcal of heat per 1 kg of evaporated sweat) [23, 24].

Sweat glands are classified into three main types: apocrine, apoeccrine, and eccrine [25, 26]. Apocrine and apoeccrine glands are limited to certain regions of the body (e.g. the axillae region) and do not become active until puberty [27–30]. Eccrine sweat glands are located across most of the body surface, are primarily responsible for thermoregulatory sweating [25], and therefore will be the focus of this review. Humans have approximately 2–3 million eccrine sweat glands and this number is fixed by approximately 2–3 years of age [31, 32]. Sweat gland density decreases with skin expansion during growth and is generally inversely proportional to body surface area (i.e. larger or more obese individuals have lower sweat gland density than their smaller counterparts) [33]. SR over the whole body is a product of the density of active sweat glands and the secretion rate per gland. At the onset of sweating (i.e. upon reaching the *T*_c_ set point), the initial response is a rapid increase in sweat gland recruitment, followed by a more gradual increase in sweat secretion per gland [31, 34–36]. Most of the intra- and interindividual variability in steady-state SR is due to differences in sweat secretion rate per gland, rather than the total number of active sweat glands or sweat gland density. With habitual activation, sweat glands show some plasticity in their size and neural/hormonal sensitivity, which in turn impact SR and sweat [Na^+^] [37–39].

Eccrine sweat glands primarily respond to thermal stimuli, particularly increased *T*_c_ [40] and also *T*_sk_ and associated increases in skin blood flow [25, 41, 42]. Sweating is mediated predominately by sympathetic cholinergic stimulation; the nerves surrounding eccrine sweat glands are nonmyelinated class C sympathetic postganglionic fibers and acetylcholine is the primary terminal neurotransmitter [25]. Eccrine glands also secrete sweat in response to adrenergic stimulation, but to a much lesser extent (approximately 10%) than that of cholinergic stimulation [29, 43]. Catecholamines, as well as other neuromodulators, such as vasoactive intestinal peptide, calcitonin gene-related peptide, and nitric oxide, have also been found to play minor roles in the neural stimulation of eccrine sweating [24, 25, 44]. In addition, eccrine sweat glands respond to non-thermal stimuli related to exercise (i.e. in the absence of or prior to changes in *T*_c_ or *T*_sk_). These non-thermal sudomotor responses are thought to be mediated by feed-forward mechanisms related to central command, the exercise pressor reflex (muscle metabo- and mechanoreceptors), osmoreceptors, and possibly baroreceptors [44, 45].

The structure of the eccrine sweat gland consists of a secretory coil and duct made up of a simple tubular epithelium. Upon stimulation, primary or precursor sweat, which is an ultrafiltrate of the plasma, is secreted by clear cells of the secretory coil. Primary sweat is nearly isotonic with blood plasma (e.g. approximately 135–145 mmol/L Na^+^, approximately 95–110 mmol/L Cl^−^, and approximately 4–5 mmol/L K^+^) [29, 46–49]. As sweat flows through the duct, Na^+^ is passively reabsorbed via epithelial Na^+^ channels (ENaCs) on the luminal membrane and actively reabsorbed via Na^+^/K^+^-ATPase transporters primarily on the basolateral membrane [25, 50]. Chloride (Cl^−^) is passively reabsorbed via the cystic fibrosis transmembrane conductance regulator (CFTR) on the luminal and basolateral ductal cell membrane [25, 51]. The result is a hypotonic (with respect to Na^+^ and Cl^−^) final sweat excreted onto the skin surface [25, 29]. Na^+^/K^+^-ATPase activity is influenced by the hormonal control of aldosterone [52]. The rate of Na^+^ and Cl^−^ reabsorption is also flow dependent, such that there is a direct relation between SR and final sweat [Na^+^] and [Cl^−^] [29]. As SR increases, the rate of Na^+^ and Cl^−^ secretion in precursor sweat increases proportionally more than the rate of Na^+^ and Cl^−^ reabsorption along the duct, and therefore leads to higher final sweat [Na^+^] and [Cl^−^] [29, 53]. The rate of sweat potassium (K^+^) loss has been reported to be indirectly related to sweat flow rate, but the underlying mechanism is unclear [29]. Nonetheless, final sweat typically has a [K^+^] similar, albeit with a slightly broader range (e.g. approximately 2–8 mmol/L), to that reported for blood plasma [54–56]. More details on the structure, function, and control of eccrine sweat glands can be found in several comprehensive reviews [19, 26, 29, 32, 44, 57].

## Measurement Techniques

### Sweating Rate

Local methods to measure SR include hygrometry and gravimetry [19]. With hygrometry, or the ventilated sweat capsule technique, dry air with a known temperature is pumped at a constant flow rate through a capsule affixed to the skin. Local SR (LSR) onto the skin surface under the capsule (approximately 1–20 cm^2^) is determined from the change in the temperature and water vapor content of the effluent compared with the influent air of the capsule [58, 59]. The ventilated capsule technique is highly reliable (coefficient of variation [CV] of 2%) [60] and is considered the reference technique for measuring LSR [60–62]. However, hygrometry can overestimate sweat flow rates because the forced ventilation and maintenance of dry skin (which facilitates sweating) under the capsule is not representative of ambient conditions in some situations (e.g. the microclimate under clothing, or exercise in humid or still air).

Gravimetric techniques involve the collection of sweat directly from the skin surface (approximately 4–100 cm^2^) using filter paper [63], absorbent patches [18, 54, 64, 65], Parafilm-M^®^ pouches [66, 67], cotton gloves/socks [68], latex gloves [68], or plastic sweat collectors [69]. With these methods, LSR is determined from the mass change of the collection system. Gravimetric techniques, particularly absorbent patches, are more practical than hygrometry for sweat-testing athletes in the field. Nonetheless, a limitation of gravimetry is that the collection system can modify the local environment and consequently alter the flow rate of sweat onto the skin surface. The lack of ventilation caused by an occlusive covering increases moisture accumulation on the skin, thereby leading to progressive blocking of sweat ducts and sweat suppression (i.e. hidromeiosis [70–75]). However, it has been proposed that hidromeiosis can be minimized by limiting the duration of the collection system on the skin and/or using patches made of a material with a high absorbent capacity [19, 64].

Two studies have compared LSR results using gravimetry versus hygrometry during steady-state cycling. Morris et al. [61] reported significantly (6–37%) greater LSR with ventilated capsules (4 cm^2^) than absorbent patches after 10 and 30 min, but there were no differences after 50 and 70 min of exercise. The results were not impacted by the anatomical site (forearm and midback) or absorbent patch size (4 cm^2^ and 36–42 cm^2^) [61]. Boisvert et al. [62] reported that LSR was significantly (27%) greater with ventilated capsules versus the Parafilm-M^®^ pouch technique in the first 20 min of exercise, but there were no differences from 20 to 60 min [62]. Several studies have also reported strong correlations between LSR at various sites across the body (using either gravimetry or hygrometry) and whole-body SR (WBSR) during exercise [64, 76, 77]. Taken together, it seems that gravimetric techniques are a reliable, portable, cost-effective alternative to hygrometry for measuring the rate of sweat appearance on the skin surface, but only after steady-state sweating has been established (e.g. 20–30 min into exercise) [61, 62].

The simplest and most accurate method to assess WBSR is via changes in nude body mass from before to after exercise [2, 4, 78]; however, corrections for non-sweat sources of body mass change should be considered. The SR calculation should be corrected for fluid intake and urine output. In addition, if athletes consume food or void their bowels during the training session, these non-sweat body mass changes should also be taken into account. It is also important to note that a portion of body mass loss during exercise occurs due to metabolic mass loss (substrate oxidation) and respiratory water loss (approximately 5–15% combined [54, 79–83]). SR calculations based on change in body mass should be corrected for metabolic mass loss and respiratory water loss, particularly when exercise lasts several hours (e.g, >2–3 h) [2], is high-intensity, and/or is performed in a cool/dry environment [79, 82, 83].

Oftentimes, nude body mass measurements are not practical in the field, thus athletes are weighed while wearing clothing; however, trapped sweat in clothing can lead to underestimations of SR. Cheuvront et al. [83] tested women wearing a racing singlet, shorts, socks, and running shoes during a 30-km treadmill run at 71% of maximal oxygen uptake in warm (30 °C dry bulb temperature, 20 °C dew point temperature) or cool (14 °C dry bulb temperature, 7 °C dew point temperature) conditions (2.1 m/s wind). When corrections were not made, trapped sweat in clothing caused an 8–10% underestimation of sweat loss (no difference between warm and cool conditions), while urine loss caused an overestimation of sweat loss by 16% (warm) or 37% (cool), and combined respiratory water loss and metabolic mass loss led to an overestimation of sweat loss by 9% (warm) or 20% (cool) [83].

For more information on determining WBSR, the reader is referred to detailed, step-by-step instructions described by Armstrong and Casa [84]. In addition, the following papers provide equations to calculate mass loss from substrate oxidation [79, 85, 86] and respiratory water [79, 85]. No equations are currently available to correct for trapped sweat in different clothing/uniform ensembles.

### Sweat [Na^+^]

The reference method for determining sweat Na^+^ loss is whole-body washdown (WBW) [84, 87, 88]. The recovery of water and Na^+^ using the WBW method has been reported to be 102 ± 2% and 99 ± 2%, respectively [87]. The WBW method is considered the most accurate measure of whole-body sweat electrolyte loss because all sweat runoff is collected and accounted for and it does not interfere with the normal evaporative sweating process; however, WBW requires a controlled laboratory setting and the mode of exercise is primarily limited to stationary cycling (for more methodological details see Armstrong and Casa [84] and Shirreffs and Maughan [87]). Thus, local methods for estimating sweat [Na^+^] are more commonly used because they are relatively simple and more practical for field studies. Local sweat [Na^+^] methods are similar to that of the gravimetric LSR techniques discussed in Sect. 3.1, i.e. filter papers [89, 90], absorbent patches [91, 92], Parafilm-M^®^ pouches [66, 67], arm bags/gloves [91, 93, 94], plastic sweat capsules [90, 95], and sweat collectors [69]. Quality-control analyses have reported a high percentage of Na^+^ recovery (99% [92]) and negligible background Na^+^ (approximately 0–3 mmol/L [92, 96]) in the absorbent patch technique. Still, local sweat [Na^+^] is usually not a valid direct surrogate for whole-body [Na^+^] due in part to the creation of a microenvironment (i.e. increased local humidity, skin wettedness, and possibly *T*_sk_). The impact of hidromeiosis on LSR and, in turn, on sweat [Na^+^], has been discussed in Sect. 3.1. In addition, leaching of electrolytes (from the stratum corneum of the skin to the local sweat sample) and/or absorption of water (from the sweat into the stratum corneum) can lead to falsely high sweat electrolyte (including Na^+^ and K^+^) concentrations from samples collected within occlusive coverings [27, 88, 97]. Because [K^+^] in final sweat is expected to be similar to plasma [K^+^] and stay relatively consistent despite changes in SR, sweat [K^+^] can be used as a quality control check of the sweat sample. If sweat [K^+^] is significantly above the normal range (e.g. >10 mmol/L), potential issues with leaching or sample evaporation/contamination may be suspected [2, 7, 18, 47, 97].

Studies conducting simultaneous local and WBW sweat [Na^+^] measurements have consistently shown that most local anatomical sites overestimate WBW sweat [Na^+^] [54, 55, 87]. The magnitude of variation between local and WBW sweat [Na^+^] depends on the anatomical site and the methodology used. For example, sweat [Na^+^] from some anatomical sites (forearm, scapula, chest, and forehead) is approximately 25–100% greater, while sweat [Na^+^] from other sites (foot, thigh, lower back) is similar to that of WBW [54, 55, 87]. This regional variation in sweat [Na^+^] can be explained in part by regional variations in LSR. Not surprisingly, inter-regional differences in LSR and sweat [Na^+^] have been reported to follow the same general pattern (e.g. forehead > chest > scapula > forearm > thigh) [55]. In addition, the arm bag or rubber glove technique tends to overestimate sweat electrolyte concentrations to a greater extent than other local methods [88, 98, 99]. Nonetheless, studies have shown that local sweat [Na^+^] is highly and significantly correlated with WBW sweat [Na^+^], thus regression equations are available to estimate WBW sweat [Na^+^] from local sweat [Na^+^] using absorbent patches [54] and Parafilm-M^®^ pouches [55]. Laboratory studies have shown that using a composite of sweat [Na^+^] from multiple regions does not improve the predictability of WBW sweat [Na^+^] [54, 55]; however, it may still be prudent to collect sweat from multiple sites when sweat testing in the field (as a measure of quality control or to have a back-up in the event that one patch falls off).

## Methodological Sources of Variability in Sweating Rate and Sweat [Na^+^]

### Sample Collection

#### Method of Sweat Stimulation

There are generally three methods by which sweating can be induced to enable sample collection: (i) pharmacological; (ii) passive thermal (heat) stress; and (iii) exercise. In studies investigating sweat gland function and responsiveness, local sweating is typically stimulated pharmacologically. This method involves the use of a small electrical current (iontophoresis) to propel charged cholinergic agonists (usually pilocarpine) transdermally to stimulate the muscarinic receptors on the sweat glands and induce sweat secretion. Pilocarpine iontophoresis was standardized by Gibson and Cooke in 1959 (Quantitative Pilocarpine Iontophoretic Test [or QPIT] method [89]). Subsequently, in 1983, a simplified version of sweat collection via pilocarpine iontophoresis was introduced (Wescor Macroduct system [100]). Pilocarpine iontophoresis is the gold-standard sweat-testing method for diagnosing cystic fibrosis (CF) [101]; however, it is important to note that the sweating response differs significantly between pharmacological and thermal and/or exercise-induced sweating. Most notably, LSR is consistently higher with exercise and thermal stress compared with pilocarpine iontophoresis [19, 102, 103]. The reason for the discrepancy could be related to the different mechanisms involved in the sudomotor response between methods. With pilocarpine-iontophoresis, sweat secretion is only induced via local cholinergic stimulation of sweat glands, whereas with exercise and/or heat stress other local (e.g. *T*_sk_, skin blood flow, adrenergic stimulation, and other neuromodulators) and central mediators (e.g. *T*_c_, central command, and exercise pressor reflex) are involved in sweat stimulation [44, 45].

Regarding the impact of sweat stimulation methodology on sweat composition, two studies have shown higher [Na^+^] and [Cl^−^] with exercise versus passive heating [98, 104]. There have been mixed results when comparing the composition of pharmacological and thermal sweat. Separate studies have reported higher [105, 106], lower [106], or similar [107] electrolyte concentrations in pharmacological versus thermal sweat, therefore more work is needed in this area. Nonetheless, it is clear that sweat tests with athletes should be conducted during exercise and in conditions (i.e. thermal environment) specific/relevant to their sport (see Table 1 for more information).

**Table 1:** Methodological sources of variability in local sweating rate and local sweat [Na^+^]

	Local SR	Local sweat [Na^+^]	Comments
*Sample collection*			
Type of sweating			
Exercise vs. pharmacological	↑	↑/↓/↔	Exercise involves central/peripheral and thermal/non-thermal mechanisms of sweating, whereas pilocarpine iontophoresis involves only peripheral cholinergic stimulation of sweat glands
Method of collection			
Local: gravimetry vs. hygrometry	↓	NA	Difference primarily early in exercise (e.g. first 20–30 min, i.e. prior to establishing steady-state sweating); microenvironment is created by both methods; gravimetry most practical in field tests
Local vs. whole-body	↑ (vs. WBSR)	↑ (vs. WB sweat [Na^+^])	Local typically overestimates WB, but varies with anatomical site
Skin surface contamination			
Scrubbing vs. light cleaning; removal of initial sweat	?	↔	Seems to impact trace minerals more than Na^+^ and K^+^
Leaching	?	↑	Leaching of electrolytes from stratum corneum into sweat and/or water from sweat into stratum corneum; can be indicated by high sweat [K^+^]
Timing			
Patch application (before vs. 20–30 min after exercise onset)	↓	↓	Lower SR at start of exercise vs. after steady-state sweating has been established
Patch removal	?	↔/?	Reported duration of patch time on skin varies from approximately 5 to approximately 90 min exercise; no differences found between 30 and 70 min in one study; more research needed
Patch saturation	↓	↓	Moisture accumulation on skin leads to hidromeiosis
	Local SR	Local sweat [Na^+^] or [Cl^−^]	Comments
*Sample storage*			
Storage temperature			3–7 days in storage; some information gleaned from the CF literature (e.g. sweat [Cl^−^])
Freezing (−80 °C)	NA	↓/?	More research needed for sweat [Na^+^]
Refrigeration (2–8 °C)	NA	↑/↔/?	More research needed for sweat [Na^+^]; CF sweat-testing guidelines recommend 4 °C for a maximum of 3 days in airtight containers
Room (21–25 °C)	NA	↑/↔/?	More research needed for sweat [Na^+^]
Incubation (32–37 °C)	NA	↑/↔/?	More research needed for sweat [Na^+^]
	Local SR	Local sweat [Na^+^]	Comments
*Sample analysis*			
Analytical technique	NA	IC < ISE < FP ≤ conductivity	General synopsis across multiple studies; more research directly comparing all techniques is needed

See text for discussion and supporting references

*CF* cystic fibrosis, *[Cl* ^*−*^ *]* chloride concentration, *FP* flame photometry, *IC* ion chromatography, *ISE* ion-specific electrode, *[K* ^*+*^ *]* potassium concentration, *[Na* ^*+*^ *]* sodium concentration, *NA* not applicable, *SR* sweating rate, *WB* whole body, *WBSR* whole-body sweating rate, ↑ indicates increase in the sweat response, ↓ indicates decrease in the sweat response, ↔ indicates no effect on the sweat response, ? indicates limited data available

#### Skin Surface Contamination and Initial Sweat

Several studies have shown that when serial samples are collected during exercise, initial sweat often has higher mineral concentrations than subsequent samples [99, 108–111]. This is likely because initial sweat mixes with minerals trapped in the sweat pore or residing in the epidermis [112–115]. The decrease in mineral concentrations observed throughout exercise is likely due to flushing of surface contamination. As Ely et al. [99] have shown, meticulously cleaning the skin (with distilled water, soap, and a surgical scrub brush) prior to collection results in stable sweat mineral concentrations during 3 h of exercise (using the Parafilm-M^®^ pouch technique on the back).

Nonetheless, it is important to note that skin surface contamination from skin desquamation and mineral residues has only been reported with trace minerals (e.g. iron [Fe], zinc [Zn], copper [Cu], magnesium [Mg], and calcium [Ca]) [99, 108–111, 114, 115]. Studies have shown no change [108] or an increase [99] in sweat [Na^+^] and [K^+^] throughout exercise, which may be explained in part by the significantly higher concentrations of Na^+^ and K^+^ in sweat (i.e. less impact on the signal-to-noise ratio) compared with trace minerals; however, more work is needed to confirm this hypothesis. Taken together, it seems prudent to clean the skin surface prior to application of the sweat collection system. This commonly includes wiping the skin surface with alcohol and/or rinsing with distilled water. Meticulous cleaning, such as shaving and/or scrubbing with a surgical brush [99], may only be necessary when conducting laboratory research, using the arm-bag technique (to remove dirt and other material under finger nails), or when measuring trace mineral concentrations in sweat (see Table 1 for more information).

#### Timing and Duration of Sweat Collection

As discussed in Sects. 2 and 3.1, LSR gradually increases from the onset of exercise until a steady state is reached. Applying absorbent patches approximately 20–30 min into the training session will provide LSR and sweat [Na^+^] results more indicative of steady-state sweating than initial sweat [61, 62]. Another important factor to consider is the timing of absorbent patch removal from the skin. A wide range in the duration of sweat collection has been reported, with some laboratory studies suggesting a maximum of 5 min [61], while approximately 15–30 min [116–119], or even up to approximately 90 min [10, 11, 13] has commonly been reported in field studies. Few studies have investigated the impact of patch adherence time on LSR or sweat [Na^+^]. Brebner and Kerslake [71] showed that a decline in sweating produced by wetting the skin with water or sweat occurs within 15 min and proceeds exponentially for at least 5 h. However, Dziedzic et al. [18] found no significant difference in forearm sweat [Na^+^] whether absorbent patches were on the skin for 30 or 70 min (although there was a non-significant 8 mmol/L or 13% increase over time). Future research is needed to determine the effects of patch adherence time (and possible interactions with LSR and patch absorbent capacity/size) on local sweat [Na^+^] and to cross-validate results against values obtained with the WBW technique (see Table 1 for more information).

#### Sample Storage

Another potential source of variability in sweat electrolyte concentrations is the method and duration of sample storage. Dziedzic et al. [18] found that, compared with immediate analysis, there was an approximately 7% decrease in sweat [Na^+^] after freezing (−80 °C) and an approximately 14% increase with refrigeration (7 °C) for 7 days. There were no significant differences in sweat [Na^+^] among samples analyzed immediately versus after being stored at room temperature (21 °C) or in an incubator (32 °C) for 7 days [18]. However, another paper reported that, after 5 days of sample storage, increases in sweat [Cl^−^] occurred to a greater extent at room temperature (21–23 °C, approximately 21–66% increase) than at refrigerated temperature (2–8 °C, approximately 3–19% increase) [120].

The primary concern associated with sample storage is evaporation; i.e. the loss of water in excess of electrolytes, leading to increases in sweat electrolyte concentrations. A few studies have measured the mass change of sweat samples to assess the impact of different storage methods on sample evaporation. In one study, 72 h of storage at room temperature (25 °C) or in refrigeration (4 °C) led to minimal sample weight change, while incubation (37 °C) led to significant sample evaporation [121]. Another study investigated sample weight changes and the impact of storage temperature (room vs. refrigeration) and the sample sealing method (Parafilm-M^®^ seal vs. plastic bag vs. none) over 5 days [120]. The authors concluded that the least evaporation occurred with refrigerated, Parafilm-M^®^ sealed samples (approximately 1% at 3 days and approximately 2% at 5 days) and the most evaporation occurred with room temperature, unsealed samples (approximately 19% at 3 days, approximately 32% at 5 days) [120]. Accordingly, sweat-testing guidelines established for the diagnosis of CF recommend samples are stored at 4 °C for a maximum of 3 days in airtight containers [122, 123]. However, the studies on which these guidelines are based did not measure sweat [Na^+^] or investigate longer durations of sample storage (i.e. >3–5 days) or the impact of temperature fluctuations (e.g. shipping across different climates). Therefore, more research is needed to determine best practices for sample transportation and sample stability related to sweat [Na^+^] (see Table 1 for more information).

#### Sample Analysis

Analytical techniques to measure sweat [Na^+^] include ion chromatography (IC), inductively-coupled plasma mass spectrometry (ICP-MS), flame photometry (FP), ion-selective electrode (ISE), and conductivity. No study, to the author’s knowledge, has directly compared all methods, but a synopsis of the literature suggests that sweat [Na^+^] is generally highest with conductivity and FP, intermediate with ISE, and lowest with IC. For example, in separate studies, conductivity produced approximately 6% higher sweat [Na^+^] values than FP [124], FP values were approximately 20% higher than ISE [18] and IC [96], and ISE values were approximately 4% [125] and approximately 10% [126] higher than IC.

Historically, FP was the analytical technique recommended when sweat [Na^+^] was used in diagnostic tests for CF [122, 127], as well as the reference method used in many sweat tests during exercise or thermal stress [13, 55, 92, 105, 118, 124, 128]; however, flame photometers have become outdated and can be difficult to service or replace [129]. Contemporary laboratory reference techniques for sweat electrolyte analysis include IC and ICP-MS, both of which require only small sample volumes and have been found to be highly sensitive, accurate, and reliable (CV approximately 1–5%) [129–131]; however, IC, and particularly ICP-MS, involves expensive equipment, labor-intensive sample preparation/analysis, and expertise [123, 129].

Techniques that are more amenable to sweat analysis in the field include ISE and conductivity. Sweat-testing guidelines for the diagnosis of CF caution against the use of conductivity because it measures the concentration of all ions (and is therefore not specific for the particular ion of interest, such as [Na^+^] or [Cl^−^]) [127, 132]. ISE techniques, while a measure of ion activity rather than a direct measure of concentration, are considered acceptable for CF diagnosis [122, 127, 133]. Studies measuring sweat [Na^+^] have found that ISE (via a compact Na^+^ analyzer; HORIBA Scientific, Irivine, CA, USA) has similar reliability to that of IC (CVs approximately 1–4% for both methods) [125, 126]. These studies also reported that compared with IC, the ISE technique produced sweat [Na^+^] values with a mean bias of approximately 2–4 mmol/L (or approximately 4–10%) [125, 126]. This small but significant discrepancy between ISE and IC could be due to the limited measurement resolution and range of ISE [125, 126, 129]. Another potential drawback of ISE is the need for larger sample volumes compared with IC and ICP-MS [126, 129]. However, investigators should weigh the shortcomings of sweat analysis in the field versus the potential error (e.g. sample evaporation/contamination) and practical inconveniences (e.g. cost, delay in obtaining results) introduced by storing and transporting samples to the laboratory for subsequent analysis. There may be situations where sweat analysis in the field is the best practice (e.g. if sample storage duration and conditions during transportation cannot be well-controlled). Nevertheless, more studies directly comparing different analytical techniques are needed (see Table 1 for more information).

## Intra/Interindividual Sources of Variability in Sweating Rate and Sweat [Na^+^]

### Intraindividual Variability

#### Day-to-Day

Even when sweat-testing methods are well-controlled, a certain amount of variability is observed within subjects. From day-to-day, WBSR has been shown to vary by approximately 5–7% [54, 134], while LSR can vary by approximately 6–17% using Parafilm-M^®^ pouches [134] and up to approximately 22% with ventilated capsules [60]. The reported day-to-day variability of WBW [Na^+^] is approximately 11–17% [54, 87] and for local sweat [Na^+^] is approximately 5–16% for absorbent patches [54] and approximately 8–12% for Parafilm-M^®^ pouches [134]. This variability in the sweating response should be taken into account when interpreting study results. For example, differences in results between tests may only have practical significance (e.g. warrant changes in fluid replacement strategy) when a change in conditions (e.g. exercise intensity, environment, equipment/clothing, etc.) elicits changes in WBSR by more than approximately 5% and sweat [Na^+^] by more than approximately 15% (see Table 2 for more information).

**Table 2:** Factors involved in the intraindividual and/or interindividual variability in sweating rate and sweat [Na^+^]

	WBSR	Local SR	Local sweat [Na^+^]	Comments
Day-to-day (CVs)	5–7%	6–22%	5–16% (WB: 11–17%)	Includes instrument variability (1–3%)
Regional differences				
Across body (% difference)	NA	200–360%	80–120%	Range includes anatomical sites typically used/accessible in field testing (back, chest, forearm, thigh, and forehead)
Contralateral sides	NA	↔	↔	Forearms and scapulas
Exercise intensity (absolute *V*O_2_)				Impacts *E*_req_
High vs. moderate vs. low	↑	↑	↑	Directly related to metabolic energy expenditure (i.e. metabolic heat production)
Environmental conditions				
Temperature (↑)	↑	↑	↑	Impacts *E*_req_; ↑ radiant heat gain and therefore ↑ *T*_c_
Solar radiation (↑)	↑	↑	↑/?	Impacts *E*_req_; ↑ radiant heat gain and therefore ↑ *T*_c_
Humidity (↑)	↑	↑	↑/?	↓ Water vapor gradient leads to ↓ evaporation of sweat, which ↑ *T*_c_ and the need for higher SR than calculated from *E*_req_, but prolonged exposure can lead to hidromeiosis and decreased SR
Wind (↑)	↓	↓	↓/?	Impacts *E*_req_; ↑ convective/evaporative heat loss and therefore ↓ *T*_c_
Body mass				
Larger vs. smaller	↑	?	?	Related to metabolic heat production and possibly sweating efficiency
Protective equipment	↑	↑	?	↓ Evaporative and radiant heat loss, ↑ metabolic heat gain and therefore ↑ *T*_c_
Sex				
Men vs. women	↑	↑	↑/↔	SR differences related to higher body mass and metabolic heat production of men, rather than sex per se; less wasteful sweating by women in humid heat
Aging				
Older vs. middle-aged vs. young adult	↓	↓	↔/?	Related to decline in fitness (and associated decline in cholinergic sensitivity), rather than aging per se
Maturation				
Pre vs. post-pubertal	↓	?	↓	Related to lower sweat gland sensitivity; SR differences in males only, suggesting testosterone may be involved (although direct evidence is lacking)
Heat acclimation	↑	↑	↓	↑ Cholinergic and aldosterone sensitivity; gland hypertrophy; ↑ slope of relation between SR and *T*_c_; ↓ *T*_c_ threshold for sweat onset
Aerobic capacity				
Higher vs. lower *V*O_2max_	↑	↑	↔/?	↑ Cholinergic sensitivity; ↑ slope of relation between SR and *T*_c_; ↓ *T*_c_ threshold for sweat onset
Hydration status				
2–3% BML vs. euhydration	↓	↓	↑/?	Hypovolemia ↓ slope of relation between SR and *T*_c_; hyperosmolality ↑ *T*_c_ threshold for sweat onset
Menstrual cycle				
Luteal vs. follicular	↔	↓	↓/↔/?	Luteal phase ↑ *T*_c_ threshold for sweat onset and ↓ slope of relation between SR and *T*_c_ (thus LSR lower at a given *T*_c_); effect lessens with heat acclimation
Dietary sodium				Studies involved 8–14 days on strictly controlled, modified diets
Change from moderate to high intake (8–9 g Na^+^)	↔	↔	↑	↓ Circulating aldosterone
Change from moderate to low intake (1–2 g Na^+^)	↔	↔	↓	↑ Circulating aldosterone
Exercise duration (↑)				
Low intensity	↔	↔	↔	Studies involved 3–7 h of exercise and low SR
High intensity	↓	↓	↓	Related to effects of hidromeiosis with prolonged heavy sweating
Race/ethnicity	↔	↔	↔	Indigenous environmental factors are more important than race or ethnicity per se. Heat habituation (lower, more efficient sweating) may occur in people indigenous to hot or tropical climates

See text for discussion and supporting references

*BML* body mass loss, *CV* coefficient of variation, *E*_*req*_ required rate of evaporation for heat balance, *NA* not applicable, *[Na* ^*+*^ *]* sodium concentration, *SR* sweating rate, *Tc* body core temperature, *VO*_*2*_ oxygen uptake, *VO*_*2max*_ maximal oxygen uptake, *WB* whole body, *WBSR* whole-body sweating rate, ↑ indicates increase in the sweating response, ↓ indicates decrease in the sweating response, ↔ indicates no effect on the sweating response, ? indicates limited data available

#### Regional

The substantial variability in LSR and local sweat [Na^+^] across the body has been extensively researched [54, 55, 64, 68, 77] and reviewed elsewhere [19]. In brief, LSR can vary by up to approximately 360% [55, 64, 68, 77] and tends to be higher on the torso versus extremities [68, 135], and, within the torso, higher posteriorly versus anteriorly, and medially versus laterally [68]. In addition, local sweat [Na^+^] across the body can vary by as much as approximately 80–120% within subjects [54, 55]. Studies report no significant bilateral differences in scapula LSR [61], forearm LSR [60, 61, 92], or forearm sweat [Na^+^] [18, 96]. There may be small differences in LSR between the dorsal and ventral forearm (7–12%) [68] but this comparison has not been well-studied. In addition, more work is needed to determine the impact of subtle differences in patch placement in a given location (e.g. proximal vs. distal forearm) on LSR and sweat [Na^+^] (see Table 2 for more information).

#### Intra- and/or Interindividual Variability

**Fig. 1:**
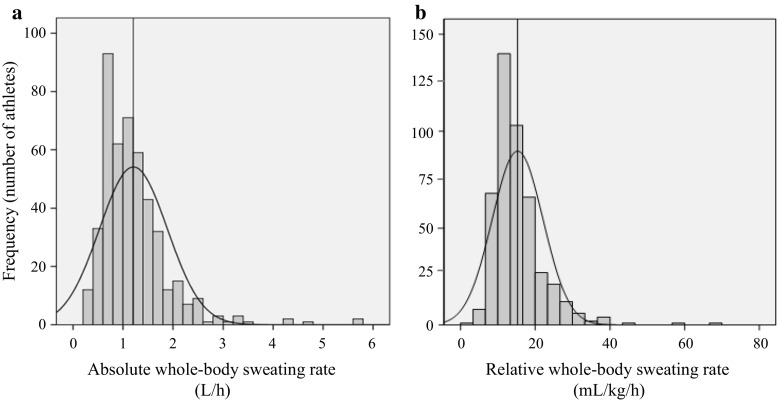
Frequency histograms of (**a**) absolute whole-body sweating rate and (**b**) relative whole-body sweating rate from 461 athletes (327 adults, 134 youth; 369 male, 92 female) of various sports (e.g. American Football, basketball, baseball, soccer, tennis, and endurance) tested during training or competition in various environmental conditions (15–50 °C, 20–79% relative humidity). The *vertical line* represents the mean value. Reproduced from Baker et al. [96], with permission

**Fig. 2:**
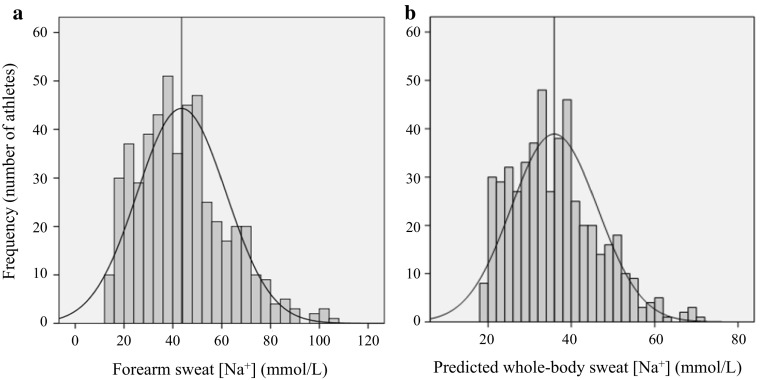
Frequency histograms of (**a**) forearm sweat sodium concentration ([Na^+^]) using the absorbent patch technique and (**b**) predicted whole-body sweat [Na^+^] from 506 athletes (367 adults, 139 youth; 404 male, 102 female) of various sports (e.g. American Football, basketball, baseball, soccer, tennis, and endurance) tested during training or competition in various environmental conditions (15–50 °C, 20–79% relative humidity). The vertical line represents the mean value. Reproduced from Baker et al. [96], with permission

Several papers have reported on the wide variability in sweat-testing results among athletes [2, 96, 136]. For example, Figs. 1 and 2 show WBSR and sweat [Na^+^], respectively, from approximately 500 athletes tested by the Gatorade Sports Science Institute [96]. As this and other studies have shown, WBSR typically ranges from approximately 0.5 to approximately 2.0 L/h [2, 24, 96]. WBSR can be >3.0 L/h [9, 15, 96, 137–141] but this is relatively rare (approximately 2% of athletes in Fig. 1a) and is usually associated with extreme circumstances (related to environment, exercise intensity, and/or large body mass) [96]. Local sweat [Na^+^] typically ranges from approximately 10 to approximately 90 mmol/L (Fig. 2a; see also Maughan and Shirreffs [46], Baker et al. [54], Patterson et al. [55], Shirreffs and Maughan [87], and Verde et al. [92]), while whole-body sweat [Na^+^] is approximately 10–70 mmol/L (predicted, Fig. 2b; measured in Baker et al. [54], Patterson et al. [55], and Shirreffs and Maughan [87]).

Many factors are involved in the variability in sweat-testing results. These factors that can lead to acute changes in the sweating response include exercise intensity, exercise duration, environmental conditions, clothing/equipment, hydration status, and circadian rhythm, while longer-term adaptations in the sweating response can occur with heat acclimation, aerobic training, and modifications in dietary sodium. Host factors, such as body mass, body composition, sex, menstrual cycle phase, maturation, aging, medications, medical conditions, and genetics can also play a role in SR and/or sweat [Na^+^] variability. The following section provides a brief summary of the factors accounting for intra- and interindividual differences in SR and sweat [Na^+^].

#### Rate of Evaporation Required for Heat Balance

According to heat-balance theory, the rate of sweat evaporation from the skin is directly determined by the evaporative requirement for heat balance (*E*_req_), which is represented by the following equation:

$$ E_{\text{req}} = M{-}W \pm (R + C + K) $$

where *M* is metabolic energy expenditure, *W* is external work, *R* is radiant heat exchange, *C* is convective heat exchange, and *K* is conductive heat exchange [142, 143]. The primary means by which the body gains heat is from metabolism (which is directly proportional to exercise intensity) and the environment, therefore these factors are also the primary determinants of sudomotor activity [142, 144]. Factors such as body size [145–148], body composition [145], sex [2, 68, 149–151], or wearing protective clothing/equipment [152–154], which (directly or indirectly) impact metabolic heat gain and/or heat loss capacity, can modify SR. Ambient temperature [142, 143], solar radiation [155–157], and wind [158, 159] impact the sudomotor response through their effects on body heat exchange with the environment (see Table 2 for more information). It is also important to note that sweating is not 100% efficient because some secreted sweat can drip from the body and not be evaporated. Therefore, in certain situations (e.g. humid environments), higher SR than calculated from *E*_req_ may be needed to satisfy the demands for cooling [160, 161].

#### Structural Differences in Sweat Glands

Some intra- and interindividual variability in SR can be explained through differences in the structure of sweat glands. Sato and colleagues have shown that glandular size (volume) can vary by as much as fivefold between individuals [25, 162], and there is a significant positive correlation between the size of isolated sweat glands and their methacholine sensitivity and maximal secretory rate [162]. Sweat gland hypertrophy and increased cholinergic sensitivity have been reported to occur with aerobic training [162] and heat acclimation [38] (see Table 2 for more information).

#### Central and Peripheral Control of the Sweating Response

As discussed in Sect. 2, sweating occurs primarily in response to increases in *T*_c_ [40]. Two important aspects of thermoregulatory sweating are the onset (i.e. *T*_c_ threshold) and sensitivity (i.e. slope of the relation between SR and the change in *T*_c_) of the sweating response to hyperthermia [163]. Shifts in the sweating temperature threshold are thought to be central (hypothalamic) in origin, whereas changes in sensitivity are peripheral (at the level of sweat glands) [40]. Several intra- and interindividual factors can modify the control of sweating [163]. For example, the enhancement of sweating with heat acclimation [37, 95, 164, 165] and aerobic training [166–169] has been associated with both an earlier onset and greater responsiveness of sweating in relation to *T*_c_ [57, 164, 170–174]. By contrast, dehydration has been shown to delay/alter the sweating response [175, 176], such that hyperosmolality increases the *T*_c_ threshold for sweating onset [177] and hypovolemia reduces sweating sensitivity [178]. The decline in SR with aging is thought to be attributed in part to factors (decline in aerobic fitness) related to decreased sensitivity of sweat glands to cholinergic stimulation [167, 179–181]. In addition, other factors, such as maturation [181–184], altitude [185–187], circadian rhythm [188, 189], and menstrual cycle [189–192] have been shown to modify the onset and/or sensitivity of the sweating response. However, it is important to note that modifications in the onset and/or sensitivity of local sweating in relation to *T*_c_ are not always associated with significant differences in overall whole-body sweat losses during exercise (e.g. across menstrual cycle phases [190, 193–195]) (see Table 2 for more information).

#### Rate of Ductal Na^+^ Reabsorption

Predominate factors involved in Na^+^ reabsorption rate in the sweat duct include ion transporter activity and sweat flow rate. Na^+^/K^+^-ATPase activity is dictated in part by aldosterone, which can be influenced by heat acclimation and dietary Na^+^. It is well known that adaptation to the heat leads to improved salt conservation through a decrease in sweat [Na^+^] [37, 95, 98, 196, 197], with the underlying mechanism related to increased sensitivity of the sweat gland to circulating aldosterone [37]. In addition, changes in dietary Na^+^ can modify aldosterone secretion, and thereby modify sweat [Na^+^] [198, 199]. Studies have shown that, compared with moderate Na^+^ intake (3–4 g/day), 8–14 days of high (8–9 g/day) or low (1–2 g/day) Na^+^ intake are associated with significant increases [128, 199] or decreases [128, 199, 200], respectively, in sweat [Na^+^]. As explained in Sect. 2, sweat flow rate and sweat [Na^+^] are directly related [53, 197], therefore acute changes in sweat flow rate, such as an increase due to more vigorous exercise [53, 197, 201] or warmer ambient temperatures [18], can also lead to increases in sweat [Na^+^].

The potential effect of other factors on sweat [Na^+^] has also been studied. Maturation may impact sweat [Na^+^] as some studies have found higher values in adults than youth, particularly among male subjects [96, 183]; however, within adults, neither sex [96, 202] nor aging [167] seem to significantly impact sweat [Na^+^]. One study [203] has reported an increase in sweat [Na^+^] with dehydration, but more research is needed to corroborate this finding and elucidate potential mechanisms. The effect of menstrual cycle phase on sweat [Na^+^] is also unclear [195, 204, 205] (see Table 2 for more information).

#### Other Factors

Another factor that can impact the sweating response is exercise duration. Prolonged heavy sweating, leading to elevated skin wettedness and hidromeiosis, can decrease SR and sweat [Na^+^] [70, 75, 135, 206]; however, studies have reported no decline in SR or sweat [Na^+^] throughout prolonged low-intensity exercise [99, 108]. Interestingly, with heat acclimation the sweat glands become resistant to hidromeiosis (because of gland hypertrophy and sweat dilution) such that higher SR can be maintained [57, 161, 207, 208].

Many investigators have reported significant differences in heat tolerance with racial/ethnic variation; however, it is thought that long-term adaptation to indigenous environmental factors is more important than race or ethnicity per se in the physiological responses to heat stress [57]. For example, heat habituation, characterized in part by lower, more efficient sweating (less dripping) may occur in people indigenous to hot or tropical climates [209–211]. For more thorough reviews on race/ethnicity and thermoregulation the reader is referred elsewhere [57, 98, 212, 213].

Finally, some medical conditions and medications can impact the sweating response. Hypohydrosis can occur as a result of pore occlusion from miliaria rubra [214, 215] or sunburn [216], as well as from medications that interfere with neural sudomotor mechanisms (e.g. anticholinergics and antidepressants such as amitriptyline) [217]. Hyperhidrosis can occur with menopause, a genetic predisposition [218], or when taking anticholinesterases and antidepressants (e.g. bupropion and venlafaxine) [217]; however, hypo- and hyperhidrosis are often localized and/or episodic and the impact on thermoregulation during exercise is not well-studied. Increased sweat [Na^+^] is associated with CF, Addison’s disease, and renal dysfunction [219]. Individuals with CF have three to five times higher sweat [Na^+^] and [Cl^−^] than normal because of a genetic absence of a functioning CFTR (two defective genes, homozygote) [220–223]. Additionally, individuals with one defective gene for CFTR (heterozygote), which is relatively common among White individuals (1/20 people), have elevated sweat [Na^+^] and [Cl^−^] [222, 224]. For more details the reader is referred to the following reviews on CF [123, 222, 225, 226] and other sweat gland disorders [217–219, 227, 228].

## Practical Recommendations

Tables 3 and 4 show a list of suggested best practices to consider when measuring WBSR and sweat [Na^+^], respectively, in athletes. These recommendations include options that allow for some methods to be adapted for the specific context of interest (e.g. laboratory vs. field-based testing). It is acknowledged that additional work is needed in some areas to corroborate or refine these best practices. Although these recommendations are intended to be used simply as a guide or educational tool, significant deviations from these methods may warrant the need for sweat testing to be repeated or, at the very least, for the results to be interpreted with caution. Furthermore, even when best practices are followed, some natural within-subject variability in WBSR and sweat [Na^+^] (e.g. approximately 5 and approximately 15% day-to-day, respectively) is still expected. For all of these reasons, categorizing athletes’ sweat water and Na^+^ losses using a low, moderate, and high categorical scheme (or something similar) and recommending a range of fluid replacement options (rather than attempting to pinpoint exact values) may be the most appropriate strategy.

**Table 3:** Best practice recommendations for measuring the whole-body sweating rate of athletes in the field

	Whole-body sweating rate
Conditions	Test in conditions (environment, intensity, season, equipment, etc.) representative of training/competition
	Conduct multiple tests within athletes to determine sweating rate in various conditions
Method	Change in body mass, preferably with athlete nude or wearing minimal clothing
Calculation	WBSR = [Body mass_PRE-EX_ – (Body mass_POST-EX_ – fluid intake_EX_ + urine output_EX_)]/exercise duration
Additional corrections	Food intake and stool loss (include in the intake and output portion of the above equation, respectively)
	Respiratory water loss and metabolic mass loss, particularly when exercise is >2–3 h, high-intensity, and/or in a dry environment
	Trapped sweat in clothing/uniform, if not obtaining nude body mass
Quality control	Take pre-exercise body mass measurement after athlete voids bladder
	Record any clothing worn during pre- and post-exercise body mass measurements
	Measure pre- and post-exercise body mass in duplicate
	Monitor fluid intake/bathroom breaks between pre- and post-exercise body mass measurements—flag data if fluid intake and urine loss are not measured
	Monitor for spitting/squirting of fluid from drink bottles—flag data if not controlled/prevented; offer a separate bottle of water if athletes want to use it for body cooling purposes (e.g. squirting on face, dumping on head, etc.)

See text for discussion and supporting references

*EX* during exercise (i.e. between pre- and post-exercise body mass measurements), *PRE-EX* pre-exercise, *POST-EX* post-exercise, *WBSR* whole-body sweating rate (typically expressed as mL/h or L/h)

**Table 4:** Best practice recommendations for measuring sweat [Na^+^] of athletes in the field using the absorbent patch technique

	Local sweat [Na^+^]
Conditions	Test during exercise (as opposed to passive heat stress or pharmacologically-induced local sweating)
	Test in conditions (environment, intensity, season, equipment, etc.) representative of training/competition
	Conduct multiple tests within athletes to determine sweat [Na^+^] in various conditions
Methods	Check for background electrolytes in collection system (e.g. patches, storage tubes, etc.)
	Anatomical location: consider site accessibility and validity compared with whole-body sweat [Na^+^] (e.g. forearm may be best suited when considering both factors)
	Clean skin immediately prior to application: alcohol, deionized/distilled water rinse, and dry with sodium-free gauze/towel
	Apply multiple patches per athlete (e.g. right and left forearm) to have a backup (e.g. in case one patch falls off)
	Apply patches 20–30 min after the onset of exercise (to establish steady-state sweating prior to sweat collection)
	Avoid hidromeiosis: prevent patch saturation by limiting patch time on skin, using patches with high absorbent capacity, and/or changing patches frequently
	Check patches for adherence to skin—flag data if patch becomes detached prematurely
	Apply multiple patches per session if expecting significant changes in factors that would impact sweating rate (exercise intensity or environment) or if conditions are conducive to whole-body hidromeiosis (e.g. prolonged intense running in humid, still air)
	Avoid cross-contamination when working with multiple athletes (e.g. use clean forcipes for each patch)
Storage	Refrigerate (e.g. approximately 4 °C) for up to approximately 3–5 days in airtight (e.g. Parafilm-M^®^ sealed) containers
Analysis	IC or ICP-MS in the laboratory; ISE in the field
	Analysis in the field recommended if sample storage duration and conditions during transportation cannot be well-controlled
Corrections	Use regression equations to predict whole-body sweat [Na^+^] from local sweat [Na^+^]
Quality control	Flag samples that meet the following criteria:
	• Sweat sample volume suggestive of saturated patch (volume depends on specific patch type and size)
	• Sweat [Na^+^] <10 mmol/L or >90 mmol/L
	• Sweat [K^+^] <2 mmol/L or >10 mmol/L

See text for discussion and supporting references

*IC* ion chromatography, *ICP-MS* inductively coupled plasma mass spectrometry, *ISE* ion-selective electrode, *[K* ^*+*^ *]* potassium concentration, *[Na* ^*+*^ *]* sodium concentration

## Conclusions

It is clear that methodological practices can be a significant source of unwanted variability in sweat-testing results. Thus, efforts should be made to understand these factors and use appropriate control and standardization when sweat-testing athletes in order to minimize errors associated with methodology. It is also important that sweat tests are interpreted in the appropriate context, i.e. (i) the results are only applicable to the specific conditions (i.e. environment, exercise intensity, etc.) in which the testing was conducted; and (ii) comparisons among sweat tests are only valid if the same methods were used. In summary, sweat testing can be a useful tool to estimate athletes’ WBSR and sweat Na^+^ loss to help guide fluid/electrolyte replacement strategies, provided that data are collected, analyzed, and interpreted appropriately.

## References

[1] Shirreffs SM, Sawka MN (2011). Fluid and electrolyte needs for training, competition, and recovery. J Sports Sci.

[2] Sawka MN, Burke LM, Eichner ER (2007). American College of Sports Medicine position stand. Exercise and fluid replacement. Med Sci Sports Exerc.

[3] Cheuvront SN, Carter R, Castellani JW (2005). Hypohydration impairs endurance exercise performance in temperate but not cold air. J Appl Physiol.

[4] Cheuvront SN, Kenefick RW (2014). Dehydration: physiology, assessment, and performance effects. Compr Physiol.

[5] Kenefick RW, Cheuvront SN, Palombo LJ (2010). Skin temperature modifies the impact of hypohydration on aerobic performance. J Appl Physiol.

[6] Coyle EF (2004). Fluid and fuel intake during exercise. J Sports Sci.

[7] Maughan RJ, Shirreffs SM (2008). Development of individual hydration strategies for athletes. Int J Sport Nutr Exerc Metab.

[8] Gibson JC, Stuart-Hill LA, Pethick W (2012). Hydration status and fluid and sodium balance in elite Canadian junior women’s soccer players in a cool environment. Appl Physiol Nutr Metab.

[9] Godek SF, Peduzzi C, Burkholder R (2010). Sweat rates, sweat sodium concentrations, and sodium losses in 3 groups of professional football players. J Athl Train.

[10] Duffield R, McCall A, Coutts AJ (2012). Hydration, sweat and thermoregulatory responses to professional football training in the heat. J Sports Sci.

[11] Kilding AE, Tunstall H, Wraith E (2009). Sweat rate and sweat electrolyte composition in international female soccer players during game specific training. Int J Sports Med.

[12] Maughan RJ, Dargavel LA, Hares R (2009). Water and salt balance of well-trained swimmers in training. Int J Sport Nutr Exerc Metab.

[13] Maughan RJ, Merson SJ, Broad NP (2004). Fluid and electrolyte intake and loss in elite soccer players during training. Int J Sport Nutr Exerc Metab.

[14] Osterberg KL, Horswill CA, Baker LB (2009). Pregame urine specific gravity and fluid intake by National Basketball Association players during competition. J Athl Train.

[15] Palmer MS, Spriet LL (2008). Sweat rate, salt loss, and fluid intake during an intense on-ice practice in elite Canadian male junior hockey players. Appl Physiol Nutr Metab.

[16] Yeargin SW, Casa DJ, Judelson DA (2010). Thermoregulatory responses and hydration practices in heat-acclimatized adolescents during preseason high school football. J Athl Train.

[17] Stofan JR, Osterberg KL, Horswill CA (2007). Daily fluid turnover during preseason training in U.S. college football. Int J Sport Nutr Exerc Metab.

[18] Dziedzic CE, Ross ML, Slater GJ (2014). Variability of Measurements of Sweat Sodium Using the regional absorbent patch method. Int J Sports Physiol Perform.

[19] Taylor NA, Machado-Moreira CA (2013). Regional variations in transepidermal water loss, eccrine sweat gland density, sweat secretion rates and electrolyte composition in resting and exercising humans. Extr Physiol Med.

[20] Gleeson M (1998). Temperature regulation during exercise. Int J Sports Med.

[21] Sawka MN, Wenger CB, Pandolf KB, Sawka MN, Gonzalez RR (1988). Physiological responses to acute exercise-heat stress. Human performance physiology and environmental medicine at terrestrial extremes.

[22] Boulant JA (1981). Hypothalamic mechanisms in thermoregulation. Fed Proc.

[23] Wenger CB (1972). Heat of evaporation of sweat: thermodynamic considerations. J Appl Physiol.

[24] Sawka MN, Leon LR, Montain SJ (2011). Integrated physiological mechanisms of exercise performance, adaptation, and maladaptation to heat stress. Compr Physiol.

[25] Sato K, Gisolfi CV, Lamb DR, Nadel ER (1993). The mechanism of eccrine sweat secretion. Exercise, heat, and thermoregulation.

[26] Sato K, Kang WH, Saga K (1989). Biology of sweat glands and their disorders: I. Normal sweat gland function. J Am Acad Dermatol.

[27] Weiner JS, Hellmann K (1960). The sweat glands. Biol Rev.

[28] Sato K, Sato F (1987). Sweat secretion by human axillary apoeccrine sweat gland in vitro. Am J Physiol.

[29] Sato K (1977). The physiology, pharmacology, and biochemistry of the eccrine sweat gland. Rev Physiol Biochem Pharmacol.

[30] Sato K, Leidal R, Sato F (1987). Morphology and development of an apoeccrine sweat gland in human axillae. Am J Physiol.

[31] Kuno Y (1938). Variations in secretory activity of human sweat glands. Lancet.

[32] Kuno Y (1956). Human perspiration.

[33] Bar-Or O, Magnusson LI, Buskirk ER (1968). Distribution of heat-activated sweat glands in obese and lean men and women. Hum Biol.

[34] Kondo N, Shibasaki M, Aoki K (2001). Function of human eccrine sweat glands during dynamic exercise and passive heat stress. J Appl Physiol.

[35] Kondo N, Takano S, Aoki K (1998). Regional differences in the effect of exercise intensity on thermoregulatory sweating and cutaneous vasodilation. Acta Physiol Scand.

[36] Randall WC (1946). Quantitation and regional distribution of sweat glands in man. J Clin Invest.

[37] Kirby CR, Convertino VA (1986). Plasma aldosterone and sweat sodium concentrations after exercise and heat acclimation. J Appl Physiol.

[38] Sato F, Owen M, Matthes R (1990). Functional and morphological changes in the eccrine sweat gland with heat acclimation. J Appl Physiol.

[39] Sato K, Dobson RL (1970). Regional and individual variations in the function of the human eccrine sweat gland. J Invest Dermatol.

[40] Nadel ER (1979). Control of sweating rate while exercising in the heat. Med Sci Sports.

[41] Wingo JE, Low DA, Keller DM (2010). Skin blood flow and local temperature independently modify sweat rate during passive heat stress in humans. J Appl Physiol.

[42] Nadel ER, Mitchell JW, Saltin B (1971). Peripheral modifications to the central drive for sweating. J Appl Physiol.

[43] Sato K (1973). Stimulation of pentose cycle in the eccrine sweat gland by adrenergic drugs. Am J Physiol.

[44] Shibasaki M, Crandall CG (2010). Mechanisms and controllers of eccrine sweating in humans. Front Biosci.

[45] Shibasaki M, Kondo N, Crandall CG (2003). Non-thermoregulatory modulation of sweating in humans. Exerc Sport Sci Rev.

[46] Maughan RJ, Shirreffs SM, Harries M, Williams C, Stanish WD, Micheli LL (1998). Fluid and electrolyte loss and replacement in exercise. Oxford textbook of sports medicine.

[47] Costill DL (1977). Sweating: its composition and effects on body fluids. Ann N Y Acad Sci.

[48] Berne RM, Levy MN (1998). Physiology.

[49] Sato K, Sato F (1990). Na^+^, K^+^, H^+^, Cl^−^, and Ca^2+^ concentrations in cystic fibrosis eccrine sweat in vivo and in vitro. J Lab Clin Med.

[50] Quinton PM (1981). Effects of some ion transport inhibitors on secretion and reabsorption in intact and perfused single human sweat glands. Pflugers Arch.

[51] Reddy MM, Quinton PM (1994). Rapid regulation of electrolyte absorption in sweat duct. J Membr Biol.

[52] Sato K, Dobson RL (1970). The effect of intracutaneous d-aldosterone and hydrocortisone on human eccrine sweat gland function. J Invest Dermatol.

[53] Buono MJ, Claros R, Deboer T (2008). Na+ secretion rate increases proportionally more than the Na+ reabsorption rate with increases in sweat rate. J Appl Physiol.

[54] Baker LB, Stofan JR, Hamilton AA (2009). Comparison of regional patch collection vs. whole body washdown for measuring sweat sodium and potassium loss during exercise. J Appl Physiol.

[55] Patterson MJ, Galloway SD, Nimmo MA (2000). Variations in regional sweat composition in normal human males. Exp Physiol.

[56] Dill DB, Hall FG, Van Beaumont W (1966). Sweat chloride concentration: sweat rate, metabolic rate, skin temperature, and age. J Appl Physiol.

[57] Taylor NA (2014). Human heat adaptation. Compr Physiol.

[58] Brengelmann GL, McKeag M, Rowell LB (1975). Use of dew-point detection for quantitative measurement of sweating rate. J Appl Physiol.

[59] Graichen H, Rascati R, Gonzalez RR (1982). Automatic dew-point temperature sensor. J Appl Physiol.

[60] Kenefick RW, Cheuvront SN, Elliott LD (2012). Biological and analytical variation of the human sweating response: implications for study design and analysis. Am J Physiol.

[61] Morris NB, Cramer MN, Hodder SG (2013). A comparison between the technical absorbent and ventilated capsule methods for measuring local sweat rate. J Appl Physiol.

[62] Boisvert P, Desruelle AV, Candas V (1997). Comparison of sweat rate measured by a pouch collector and a hygrometric technique during exercise. Can J Appl Physiol.

[63] Ohara K (1968). Heat tolerance and sweating type. Nagoya Med J.

[64] Havenith G, Fogarty A, Bartlett R (2008). Male and female upper body sweat distribution during running measured with technical absorbents. Eur J Appl Physiol.

[65] Ogata K (1935). Functional variations in human sweat glands, with remarks upon the regional difference of the amount of sweat. J Orient Med.

[66] Brisson GR, Boisvert P, Peronnet F (1991). A simple and disposable sweat collector. Eur J Appl Physiol.

[67] Boisvert P, Nakamura K, Shimai S (1993). A modified, local sweat collector for warm and humid conditions. Eur J Appl Physiol.

[68] Smith CJ, Havenith G (2012). Body mapping of sweating patterns in athletes: a sex comparison. Med Sci Sports Exerc.

[69] Cole DE, Boucher MJ (1986). Use of a new sample-collection device (Macroduct) in anion analysis of human sweat. Clin Chem.

[70] Collins KJ, Weiner JS (1962). Observations on arm-bag suppression of sweating and its relationship to thermal sweat-gland ‘fatigue’. J Physiol.

[71] Brebner DF, Kerslake DM (1964). The time course of the decline in sweating produced by wetting the skin. J Physiol.

[72] Randall WC, Peiss CN (1957). The relationship between skin hydration and the suppression of sweating. J Invest Dermatol.

[73] Candas V, Libert JP, Vogt JJ (1983). Sweating and sweat decline of resting men in hot humid environments. Eur J Appl Physiol.

[74] Candas V, Libert JP, Vogt JJ (1979). Human skin wettedness and evaporative efficiency of sweating. J Appl Physiol.

[75] Candas V, Libert JP, Vogt JJ (1980). Effect of hidromeiosis on sweat drippage during acclimation to humid heat. Eur J Appl Physiol.

[76] Bain AR, Deren TM, Jay O (2011). Describing individual variation in local sweating during exercise in a temperate environment. Eur J Appl Physiol.

[77] Smith CJ, Havenith G (2011). Body mapping of sweating patterns in male athletes in mild exercise-induced hyperthermia. Eur J Appl Physiol.

[78] Armstrong LE (2007). Assessing hydration status: the elusive gold standard. J Am Coll Nutr.

[79] Maughan RJ, Shirreffs SM, Leiper JB (2007). Errors in the estimation of hydration status from changes in body mass. J Sports Sci.

[80] Rogers G, Goodman C, Rosen C (1997). Water budget during ultra-endurance exercise. Med Sci Sports Exerc.

[81] Cheuvront SN, Montain SJ, Goodman DA (2007). Evaluation of the limits to accurate sweat loss prediction during prolonged exercise. Eur J Appl Physiol.

[82] Cheuvront S, Haymes EM (2001). Ad libitum fluid intakes and thermoregulatory responses of female distance runner in three environments. J Sports Sci.

[83] Cheuvront SN, Haymes EM, Sawka MN (2002). Comparison of sweat loss estimates for women during prolonged high-intensity running. Med Sci Sports Exerc.

[84] Armstrong LE, Casa DJ (2009). Methods to evaluate electrolyte and water turnover of athletes. Athl Train Sports Health Care.

[85] Mitchell JW, Nadel ER, Stolwijk JA (1972). Respiratory weight losses during exercise. J Appl Physiol.

[86] Brooks G, Mercier J (1994). Balance of carbohydrate and lipid utilization during exercise: the “crossover” concept. J Appl Physiol.

[87] Shirreffs SM, Maughan RJ (1997). Whole body sweat collection in humans: an improved method with preliminary data on electrolyte content. J Appl Physiol.

[88] Van Heyningen R, Weiner JS (1952). A comparison of arm-bag sweat and body sweat. J Physiol.

[89] Gibson LE, Cooke RE (1959). A test for concentration of electrolytes in sweat in cystic fibrosis of the pancreas utilizing pilocarpine by iontophoresis. Pediatrics.

[90] Lemon PW, Yarasheski KE, Dolny DG (1986). Validity/reliability of sweat analysis by whole-body washdown vs. regional collections. J Appl Physiol.

[91] Costa F, Calloway DH, Margen S (1969). Regional and total body sweat composition of men fed controlled diets. Am J Clin Nutr.

[92] Verde T, Shephard RJ, Corey P (1982). Sweat composition in exercise and in heat. J Appl Physiol.

[93] Consolazio CF, Matoush LO, Nelson RA (1963). Excretion of sodium, potassium, magnesium and iron in human sweat and the relation of each to balance and requirements. J Nutr.

[94] Johnson RE, Pitts GC, Consolazio FC (1944). Factors influencing chloride concentration in human sweat. Am J Physiol.

[95] Allan JR, Wilson CG (1971). Influence of acclimatization on sweat sodium concentration. J Appl Physiol.

[96] Baker LB, Barnes KA, Anderson ML (2015). Normative data for regional sweat sodium concentration and whole-body sweating rate in athletes. J Sports Sci.

[97] Weschler LB (2008). Sweat electrolyte concentrations obtained from within occlusive coverings are falsely high because sweat itself leaches skin electrolytes. J Appl Physiol.

[98] Robinson S, Robinson AH (1954). Chemical composition of sweat. Physiol Rev.

[99] Ely MR, Kenefick RW, Cheuvront SN (2011). Surface contamination artificially elevates initial sweat mineral concentrations. J Appl Physiol.

[100] Webster HL (1983). Laboratory diagnosis of cystic fibrosis. Crit Rev Clin Lab Sci.

[101] Mishra A, Greaves R, Massie J (2005). The relevance of sweat testing for the diagnosis of cystic fibrosis in the genomic era. Clin Biochem Rev.

[102] Hjortskov N, Jepsen LT, Nielsen B (1995). Pilocarpine iontophoresis test: an index of physiological sweat secretion?. Clin Physiol.

[103] Vimieiro-Gomes AC, Magalhaes FC, Amorim FT (2005). Comparison of sweat rate during graded exercise and the local rate induced by pilocarpine. Braz J Med Biol Res.

[104] Ikai K, Sato K, Sugiyama K (1969). Comparison of human sweat electrolyte concentration in mental, thermal and exercise perspiration. Nagoya Med J.

[105] Sato K, Feibleman C, Dobson RL (1970). The electrolyte composition of pharmacologically and thermally stimulated sweat: a comparative study. J Invest Dermatol.

[106] Schwachman H, Antonowicz I (1962). The sweat test in cystic fibrosis. Ann N Y Acad Sci.

[107] di Sant’Agnese PA, Powell GF (1962). The eccrine sweat defect in cystic fibrosis of the pancreas. Ann NY Acad Sci.

[108] Montain SJ, Cheuvront SN, Lukaski HC (2007). Sweat mineral-element responses during 7 h of exercise-heat stress. Int J Sport Nutr Exerc Metab.

[109] Tipton K, Green NR, Haymes EM (1993). Zinc loss in sweat of athletes exercising in hot and neutral temperatures. Int J Sport Nutr.

[110] DeRuisseau KC, Cheuvront SN, Haymes EM (2002). Sweat iron and zinc losses during prolonged exercise. Int J Sport Nutr Exerc Metab.

[111] Ely MR, Kenefick RW, Cheuvront SN (2013). The effect of heat acclimation on sweat microminerals: artifact of surface contamination. Int J Sport Nutr Exerc Metab.

[112] Brown H (1927). The mineral content of human skin. J Biol Chem.

[113] Brown H (1926). Mineral content of human, dog, and rabbit skin. J Biol Chem.

[114] Brune M, Magnusson B, Persson H (1986). Iron losses in sweat. Am J Clin Nutr.

[115] Hohnadel DC, Sunderman FW, Nechay MW (1973). Atomic absorption spectrometry of nickel, copper, zinc, and lead in sweat collected from healthy subjects during sauna bathing. Clin Chem.

[116] Palmer MS, Logan HM, Spriet LL (2010). On-ice sweat rate, voluntary fluid intake, and sodium balance during practice in male junior ice hockey players drinking water or a carbohydrate-electrolyte solution. Appl Physiol Nutr Metab.

[117] Pahnke MD, Trinity JD, Zachwieja JJ (2010). Serum sodium concentration changes are related to fluid balance and sweat sodium loss. Med Sci Sports Exerc.

[118] Shirreffs SM, Aragon-Vargas LF, Chamorro M (2005). The sweating response of elite professional soccer players to training in the heat. Int J Sports Med.

[119] Stofan JR, Zachwieja JJ, Horswill CA (2005). Sweat and sodium losses in NCAA football players: a precursor to heat cramps?. Int J Sport Nutr Exerc Metab.

[120] Bergeron J, Bachmann LM, Miller WG (2011). Influence of sample storage conditions on sweat chloride results. Clin Chem.

[121] Jones PM, McCullom V, Moore G (2008). How soon is “promptly”? Storage and handling of samples for the analysis of sweat chloride. Lab Med.

[122] Green A, Kirk J, Guidelines Development G (2007). Guidelines for the performance of the sweat test for the diagnosis of cystic fibrosis. Ann Clin Biochem.

[123] Collie JT, Massie RJ, Jones OA (2014). Sixty-five years since the New York heat wave: advances in sweat testing for cystic fibrosis. Pediatr Pulm.

[124] Boisvert P, Candas V (1994). Validity of the Wescor’s sweat conductivity analyzer for the assessment of sweat electrolyte concentrations. Eur J Appl Physiol.

[125] Goulet ED, Dion T, Myette-Cote E (2012). Validity and reliability of the Horiba C-122 compact sodium analyzer in sweat samples of athletes. Eur J Appl Physiol.

[126] Baker LB, Ungaro CT, Barnes KA (2014). Validity and reliability of a field technique for sweat Na+ and K+ analysis during exercise in a hot-humid environment. Physiol Rep.

[127] Party ASTW, Coakley J, Scott S (2006). Australian guidelines for the performance of the sweat test for the diagnosis of cystic fibrosis: report from the AACB sweat testing working party. Clin Biochem Rev.

[128] Armstrong LE, Costill DL, Fink WJ (1985). Effects of dietary sodium on body and muscle potassium content during heat acclimation. Eur J Appl Physiol.

[129] Doorn J, Storteboom TT, Mulder AM (2015). Ion chromatography for the precise analysis of chloride and sodium in sweat for the diagnosis of cystic fibrosis. Ann Clin Biochem.

[130] Pullan NJ, Thurston V, Barber S (2013). Evaluation of an inductively coupled plasma mass spectrometry method for the analysis of sweat chloride and sodium for use in the diagnosis of cystic fibrosis. Ann Clin Biochem.

[131] Boulyga SF, Heilmann J, Prohaska T (2007). Development of an accurate, sensitive, and robust isotope dilution laser ablation ICP-MS method for simultaneous multi-element analysis (chlorine, sulfur, and heavy metals) in coal samples. Anal Bioanal Chem.

[132] LeGrys VA, Yankaskas JR, Quittell LM, Cystic Fibrosis F (2007). Diagnostic sweat testing: the Cystic Fibrosis Foundation guidelines. J Pediatr.

[133] Hulstein JJ, van t Sant P (2011). Sweat analysis using indirect ion-selective electrode on the routine chemistry analyser meets UK guidelines. Ann Clin Biochem.

[134] Hayden G, Milne HC, Patterson MJ (2004). The reproducibility of closed-pouch sweat collection and thermoregulatory responses to exercise-heat stress. Eur J Appl Physiol.

[135] Sawka MN, Wenger CB, Pandolf KB, Blatteis CM, Fregly MJ (1996). Thermoregulatory responses to acute exercise-heat stress and heat acclimation. Handbook of physiology, section 4: environmental physiology.

[136] Burke LM, Hawley JA (1997). Fluid balance in team sports. Guidelines for optimal practices. Sports Med.

[137] Bergeron MF (2003). Heat cramps: fluid and electrolyte challenges during tennis in the heat. J Sci Med Sport.

[138] Zetou E, Giatsis G, Mountaki F (2008). Body weight changes and voluntary fluid intakes of beach volleyball players during an official tournament. J Sci Med Sport.

[139] Brown D, Winter EM, Lees A, Maynard I, Hughes M, Reilly T (1998). Fluid loss during international standard match-play in squash. Science and racquet sports II.

[140] Armstrong LE, Hubbard RW, Jones BH (1986). Preparing Alberto Salazar for the heat of the Olympic marathon. Phys Sports Med.

[141] Godek SF, Bartolozzi AR, Godek JJ (2005). Sweat rate and fluid turnover in American football players compared with runners in a hot and humid environment. Br J Sports Med.

[142] Gagnon D, Jay O, Kenny GP (2013). The evaporative requirement for heat balance determines whole-body sweat rate during exercise under conditions permitting full evaporation. J Physiol.

[143] Nielsen M (1938). Die Regulation der Korpertemperatur beiMuskelarbeit. Skand Arch Physiol.

[144] Cramer MN, Jay O (2015). Explained variance in the thermoregulatory responses to exercise: the independent roles of biophysical and fitness/fatness-related factors. J Appl Physiol.

[145] Buresh R, Berg K, Noble J (2005). Heat production and storage are positively correlated with measures of body size/composition and heart rate drift during vigorous running. Res Q Exerc Sport.

[146] Marino FE, Mbambo Z, Kortekaas E (2000). Advantages of smaller body mass during distance running in warm, humid environments. Pflug Arch.

[147] Dennis SC, Noakes TD (1999). Advantages of a smaller bodymass in humans when distance running in warm humid conditions. Eur J Appl Physiol.

[148] Deren TM, Coris EE, Bain AR (2012). Sweating is greater in NCAA football linemen independently of heat production. Med Sci Sports Exerc.

[149] Avellini BA, Shapiro Y, Pandolf KB (1980). Physiological responses of men and women to prolonged dry heat exposure. Aviat Space Environ Med.

[150] Shapiro Y, Pandolf KB, Avellini BA (1980). Physiological responses of men and women to humid and dry heat. J Appl Physiol.

[151] Inoue Y, Ichinose-Kuwahara T, Funaki C (2014). Sex differences in acetylcholine-induced sweating responses due to physical training. J Physiol Anthropol.

[152] Armstrong LE, Johnson EC, Casa DJ (2010). The American football uniform: uncompensable heat stress and hyperthermic exhaustion. J Athl Train.

[153] McLellan TM, Daanen HA, Cheung SS (2013). Encapsulated environment. Compr Physiol.

[154] Mathews DK, Fox EL, Tanzi D (1969). Physiological responses during exercise and recovery in a football uniform. J Appl Physiol.

[155] Gonzalez RR, Cheuvront SN, Ely BR (2012). Sweat rate prediction equations for outdoor exercise with transient solar radiation. J Appl Physiol.

[156] Nielsen B, Kassow K, Aschengreen FE (1988). Heat balance during exercise in the sun. Eur J Appl Physiol.

[157] Gagge AP, Hardy JD (1967). Thermal radiation exchange of the human by partitional calorimetry. J Appl Physiol.

[158] Adams WC, Mack GW, Langhans GW (1992). Effects of varied air velocity on sweating and evaporative rates during exercise. J Appl Physiol.

[159] Shaffrath JD, Adams WC (1984). Effects of airflow and work load on cardiovascular drift and skin blood flow. J Appl Physiol.

[160] Shapiro Y, Pandolf KB, Goldman RF (1982). Predicting sweat loss response to exercise, environment and clothing. Eur J Appl Physiol.

[161] Sawka MN, Castellani JW, Cheuvront SN, Farrell PA, Joyner MJ, Caiozzo VJ (2012). Physiologic systems and their responses to conditions of heat and cold. ACSM’s advanced exercise physiology.

[162] Sato K, Sato F (1983). Individual variations in structure and function of human eccrine sweat gland. Am J Physiol.

[163] Armstrong LE, Maresh CM (1998). Effects of training, environment, and host factors on the sweating response to exercise. Int J Sports Med.

[164] Pandolf KB, Cadarette BS, Sawka MN (1988). Thermoregulatory responses of middle-aged and young men during dry-heat acclimation. J Appl Physiol.

[165] Wenger CB, Pandolf KB, Sawka MN, Gonzalez RR (1988). Human heat acclimatization. Human performance physiology and environmental medicine at terrestrial extremes.

[166] Greenleaf JE, Castle BL, Ruff WK (1972). Maximal oxygen uptake, sweating and tolerance to exercise in the heat. Int J Biometeorol.

[167] Inoue Y, Havenith G, Kenney WL (1999). Exercise- and methylcholine-induced sweating responses in older and younger men: effect of heat acclimation and aerobic fitness. Int J Biometeorol.

[168] Buono MJ, McKenzie BK, Kasch FW (1991). Effects of ageing and physical training on the peripheral sweat production of the human eccrine sweat gland. Age Ageing.

[169] Buono MJ, Sjoholm NT (1988). Effect of physical training on peripheral sweat production. J Appl Physiol.

[170] Roberts MF, Wenger CB, Stolwijk JA (1977). Skin blood flow and sweating changes following exercise training and heat acclimation. J Appl Physiol.

[171] Nadel ER, Pandolf KB, Roberts MF (1974). Mechanisms of thermal acclimation to exercise and heat. J Appl Physiol.

[172] Lee JB, Kim TW, Min YK (2014). Long distance runners present upregulated sweating responses than sedentary counterparts. PLoS One.

[173] Baum E, Bruck K, Schwennicke HP (1976). Adaptive modifications in the thermoregulatory system of long-distance runners. J Appl Physiol.

[174] Buono MJ, White CS, Connolly KP (1992). Cholinergic sensitivity of the eccrine sweat gland in trained and untrained men. J Dermatol Sci.

[175] Sawka MN, Young AJ, Francesconi RP, Pandolf KB (1985). Thermoregulatory and blood responses during exercise at graded hypohydration levels. J Appl Physiol.

[176] Montain SJ, Latzka WA, Sawka MN (1995). Control of thermoregulatory sweating is altered by hydration level and exercise intensity. J Appl Physiol.

[177] Fortney SM, Wenger CB, Bove JR (1984). Effect of hyperosmolality on control of blood flow and sweating. J Appl Physiol.

[178] Fortney SM, Nadel ER, Wenger CB (1981). Effect of blood volume on sweating rate and body fluids in exercising humans. J Appl Physiol.

[179] Kenney WL, Fowler SR (1988). Methylcholine-activated eccrine sweat gland density and output as a function of age. J Appl Physiol.

[180] Kenney WL, Munce TA (2003). Invited review: aging and human temperature regulation. J Appl Physiol.

[181] Inbar O, Morris N, Epstein Y (2004). Comparison of thermoregulatory responses to exercise in dry heat among prepubertal boys, young adults and older males. Exp Physiol.

[182] Rowland T (2008). Thermoregulation during exercise in the heat in children: old concepts revisited. J Appl Physiol.

[183] Meyer F, Bar-Or O, MacDougall D (1992). Sweat electrolyte loss during exercise in the heat: effects of gender and maturation. Med Sci Sports Exerc.

[184] Falk B, Bar-Or O, Calvert R (1992). Sweat gland response to exercise in the heat among pre-, mid-, and late-pubertal boys. Med Sci Sports Exerc.

[185] Kolka MA, Stephenson LA, Rock PB (1987). Local sweating and cutaneous blood flow during exercise in hypobaric environments. J Appl Physiol.

[186] DiPasquale DM, Kolkhorst FW, Nichols JF (2002). Effect of acute normobaric hypoxia on peripheral sweat rate. High Alt Med Biol.

[187] Kacin A, Golja P, Eiken O (2007). The influence of acute and 23 days of intermittent hypoxic exposures on the exercise-induced forehead sweating response. Eur J Appl Physiol.

[188] Wenger CB, Roberts MF, Stolwijk JA (1976). Nocturnal lowering of thresholds for sweating and vasodilation. J Appl Physiol.

[189] Stephenson LA, Kolka MA (1985). Menstrual cycle phase and time of day alter reference signal controlling arm blood flow and sweating. Am J Physiol.

[190] Inoue Y, Tanaka Y, Omori K (2005). Sex- and menstrual cycle-related differences in sweating and cutaneous blood flow in response to passive heat exposure. Eur J Appl Physiol.

[191] Kuwahara T, Inoue Y, Taniguchi M (2005). Effects of physical training on heat loss responses of young women to passive heating in relation to menstrual cycle. Eur J Appl Physiol.

[192] Kolka MA, Stephenson LA (1989). Control of sweating during the human menstrual cycle. Eur J Appl Physiol.

[193] Horvath SM, Drinkwater BL (1982). Thermoregulation and the menstrual cycle. Aviat Space Environ Med.

[194] Sunderland C, Nevill M (2003). Effect of the menstrual cycle on performance of intermittent, high-intensity shuttle running in a hot environment. Eur J Appl Physiol.

[195] Janse DEJXA, Thompson MW, Chuter (2012). Exercise performance over the menstrual cycle in temperate and hot, humid conditions. Med Sci Sports Exerc.

[196] Chinevere TD, Kenefick RW, Cheuvront SN (2008). Effect of heat acclimation on sweat minerals. Med Sci Sports Exerc.

[197] Buono MJ, Ball KD, Kolkhorst FW (2007). Sodium ion concentration vs. sweat rate relationship in humans. J Appl Physiol.

[198] Sigal CB, Dobson RL (1968). The effect of salt intake on sweat gland function. J Invest Dermatol.

[199] Allsopp AJ, Sutherland R, Wood P (1998). The effect of sodium balance on sweat sodium secretion and plasma aldosterone concentration. Eur J Appl Physiol.

[200] Hargreaves M, Morgan TO, Snow R (1989). Exercise tolerance in the heat on low and normal salt intakes. Clin Sci.

[201] Yoshida T, Shin-ya H, Nakai S (2006). Genomic and non-genomic effects of aldosterone on the individual variation of the sweat Na+ concentration during exercise in trained athletes. Eur J Appl Physiol.

[202] Morimoto T, Slabochova Z, Naman RK (1967). Sex differences in physiological reactions to thermal stress. J Appl Physiol.

[203] Morgan RM, Patterson MJ, Nimmo MA (2004). Acute effects of dehydration on sweat composition in men during prolonged exercise in the heat. Acta Physiol Scand.

[204] Lieberman J (1966). Cyclic fluctuation of sweat electrolytes in women. JAMA.

[205] Taylor JR, Sato K, Morris D (1969). Variation in sweat gland function during the menstrual cycle. J Invest Dermatol.

[206] Brown WK, Sargent F (1965). Hidromeiosis. Arch Environ Health.

[207] Ogawa T, Asayama M, Miyagawa T (1982). Effects of sweat gland training by repeated local heating. Jpn J Physiol.

[208] Yoshimura H (1960). Acclimatization to heat and cold. Essential problems in climatic physiology.

[209] Hori S (1995). Adaptation to heat. Jpn J Physiol.

[210] Candas V, Dejours P (1987). Adaptation to extreme environments. Thermophysiological changes in man during humid heat acclimation. Comparative physiology of environmental adaptations.

[211] Bae JS, Lee JB, Matsumoto T (2006). Prolonged residence of temperate natives in the tropics produces a suppression of sweating. Pflug Arch.

[212] Taylor NAS (2006). Ethnic differences in thermoregulation: genotypic versus phenotypic heat adaptation. J Therm Biol.

[213] Lambert MI, Mann T, Dugas JP (2008). Ethnicity and temperature regulation. Med Sport Sci.

[214] Pandolf KB, Griffin TB, Munro EH (1980). Heat intolerance as a function of percent of body surface involved with miliaria rubra. Am J Physiol.

[215] Pandolf KB, Griffin TB, Munro EH (1980). Persistence of impaired heat tolerance from artificially induced miliaria rubra. Am J Physiol.

[216] Pandolf KB, Gange RW, Latzka WA (1992). Human thermoregulatory responses during heat exposure after artificially induced sunburn. Am J Physiol.

[217] Cheshire WP, Fealey RD (2008). Drug-induced hyperhidrosis and hypohidrosis: incidence, prevention and management. Drug Saf.

[218] Schlereth T, Dieterich M, Birklein F (2009). Hyperhidrosis—causes and treatment of enhanced sweating. Dtsch Arztebl Int.

[219] Cheshire WP, Freeman R (2003). Disorders of sweating. Semin Neurol.

[220] Rowe SM, Miller S, Sorscher EJ (2005). Cystic fibrosis. N Engl J Med.

[221] Goodman BE, Percy WH (2005). CFTR in cystic fibrosis and cholera: from membrane transport to clinical practice. Adv Physiol Educ.

[222] Eichner ER (2008). Genetic and other determinants of sweat sodium. Curr Sports Med Rep.

[223] Reddy MM, Quinton PM (2003). Functional interaction of CFTR and ENaC in sweat glands. Pflug Arch.

[224] Farrell PM, Koscik RE (1996). Sweat chloride concentrations in infants homozygous or heterozygous for F508 cystic fibrosis. Pediatrics.

[225] Quinton PM (1999). Physiological basis of cystic fibrosis: a historical perspective. Physiol Rev.

[226] Quinton PM (2007). Cystic fibrosis: lessons from the sweat gland. Physiology.

[227] Sato K, Kang WH, Saga K (1989). Biology of sweat glands and their disorders: II. Disorders of sweat gland function. J Am Acad Dermatol.

[228] Wenzel FG, Horn TD (1998). Nonneoplastic disorders of the eccrine glands. J Am Acad Dermatol.

